# Automated Method of Extracting Urban Roads Based on Region Growing from Mobile Laser Scanning Data

**DOI:** 10.3390/s19235262

**Published:** 2019-11-29

**Authors:** Peng Li, Ruisheng Wang, Yanxia Wang, Ge Gao

**Affiliations:** 1 School of Geographic Information and Tourism, Chuzhou University, No. 1 Huifeng West Road, Chuzhou 239000, China; lipeng@chzu.edu.cn (P.L.); surveymapping@126.com (Y.W.); cicga635457049@163.com (G.G.); 2 Department of Geomatics Engineering, University of Calgary, 2500 University Drive NW, Calgary, AB T2N 1N4, Canada; 3 School of Geographical Sciences, Guangzhou University, No. 230, Waihuan West Road, Guangzhou 510006, China

**Keywords:** point cloud, mobile laser scanning, region growing, road extraction, tangent plane

## Abstract

With the rapid development of three-dimensional point cloud acquisition from mobile laser scanning systems, the extraction of urban roads has become a major research focus. Although it has great potential for digital image processing, the extraction of roads using the region growing approach is still in its infancy. We propose an automated method of urban road extraction based on region growing. First, an initial seed is chosen under constraints relating to the Gaussian curvature, height and number of neighboring points, which ensures that the initial seed is located on a road. Then, the growing condition is determined by the angle threshold of the tangent plane of the seed point. Then, new seeds are selected based on the identified road points and their curvature. The method also includes a strategy for dealing with multiple discontinuous roads in a dataset. The result shows that the method can not only achieve high accuracy in urban road extraction but is also stable and robust.

## 1. Introduction

Urban roads, as a major element of a city’s infrastructure, have always been important in the life of city dwellers, because people’s business and pleasure can be connected through them [1]. In addition, roads can provide contextual clues for recognizing other elements, such as road signs, road markings, and cars. Thus, the accurate extraction of roads can provide important support for road surveying, autonomous vehicle navigation, and other applications [2].

However, the traditional means of extracting urban road information are not only inefficient, but also expensive [3]. In recent years, because it employs a non-contact approach and has a high speed and accuracy, three-dimensional (3D) laser scanning has become one of the most effective measurement techniques, and using 3D point cloud data to extract urban features has become popular in research [4]. The sources of 3D point cloud data include airborne laser scanning (ALS), terrestrial laser scanning (TLS) and mobile laser scanning (MLS). Among these, MLS, which is faster than TLS in terms of acquisition speed and higher than ALS in terms of data resolution, has been widely used in route planning, road design and road surveying [5].

Currently, there are many ways to extract road pavement from a 3D point cloud. Guan divided them into five categories: the geometric shape of a road, the use of the geometric features and LiDAR data characteristics of a road, the data format, the use of classification methods, and external data sources [6]. In terms of technical independence, there are two principal types of method. One involves extracting 3D road surface points using road structure representation with only the point cloud data, such as cluster analysis [7], Hough transform [8], RANdom SAmple Consensus (RANSAC) [9] and weighted least squares linear fit [10]. These methods can achieve an accuracy of more than 90%, but they are quite time consuming and computationally intensive. In order to improve their computational efficiency, many other new methods have been proposed. A method based on the scan line [11] has been proven to be effective, which detects the slope and elevation of a local terrain along a series of scan lines. However, the method has some restrictions, because the point clouds lack the characteristics of a scan line order [4]. Others extract roads by detecting road edges, for example, Yoon and Crane [12] used the slope and standard deviation to estimate the road edge; Ibrahim and Lichti [13] applied the derivative of the Gaussian function to mobile LiDAR points to extract the edge of the road; and Yang [14] modeled the curb based on elevation difference, point density and slope change. These methods can improve efficiency, but the steps of most methods are complicated, and some need other information. The second type of method uses other information to enhance road recognition, such as intensity [15,16], point color [12], trajectory data [17], road properties [18], and other types of data [19,20] (video cameras, ASL, TSL, etc.). Both the accuracy and efficiency of these methods are optimized. However, if the original point cloud datasets have only spatial coordinates, all of them will be invalid. When they do have only spatial coordinates, an effective automated method for extracting road pavement from MLS becomes important. In recent years, many scholars have begun to apply deep learning to the extraction and identification of roads. Balado [21] and Yao et al. [22,23] combined deep learning and semantic segmentation to identify road environments. However, deep learning requires a lot of time to train the sample.

Region growing is mainly used for image segmentation [24] and edge detection [25]. The basic concept of region growing is the merging of similar points by determining the similarity of attributes between seeds and their neighboring points, with continued iteration until all points are processed. Since the method of region growing was proposed, it has been used for road extraction from multiple image data [26,27,28]. After 3D point cloud technology matured, region growing began to be widely used in point cloud segmentation [29,30] and the extraction of urban elements [31,32]. Nonetheless, region growing is currently less used in road extraction using MLS data. Na [33] proposed a method of extracting roads based on region growing, but it requires vehicle pose information [34,35].

In order to solve the problem of creating automatic road extraction technology when there is only spatial coordinate information in the MLS data, in this paper, we propose an automated method of road extraction based on region growing. First, an initial seed is chosen under the constraints of the curvature, height and number of neighboring points, which ensures that the initial seed is located on the road. Then, the road points are evaluated in terms of the angle threshold of the tangent plane of the seed. Then, new seeds are selected based on the identified road points and their curvature. This method also includes a strategy for dealing with multiple discontinuous roads in a dataset. The results show that the method can not only achieve a high accuracy in urban road extraction but is also robust.

There are five sections in this paper. The first chapter introduces the research background and status. Section 2 expounds the principle of this method, including the initial seed selection, road cluster growth judgment, new seed selection, and the strategy of discontinuous roads. Section 3 describes the experimental data, ground truth and accuracy assessment. Section 4 provides the experimental results and analysis, and Section 5 presents the conclusion.

## 2. Urban Road Extraction Using Region Growing

### 2.1. Method of Region Growing

**Figure 1:**
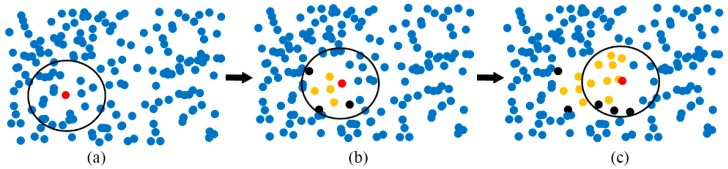
Principle of region growing. The red point is the initial seed; the yellow points are in the same cluster; the black are not in the same cluster; and the blue are the undetermined points. (**a**) Initial seed and neighborhood; (**b**) judgment of neighboring points; (**c**) the process of region growing.

Region growing merges points that are sufficiently close in terms of smoothness constraint, and each type of merged point is considered to be in the same cluster. The steps of the algorithm are as follows (Figure 1). First, the initial seed point is chosen, and the Ks of its nearest neighbors are found. Second, it is determined whether each neighbor belongs to the same cluster as the seed. If the neighbor belongs to the same class, it is added to the cluster of the seed and marked as classified. If not, the point is returned to the unclassified data. Third, it is determined whether each classified point can be used as a new seed point. If so, the point is added to the seed set, and the steps of finding the seed and its cluster are repeated, until no more new points can be added. Finally, the above steps are repeated for a new cluster, until all points are classified. Figure 1 illustrates the process of region growing. The red point is the seed; the blue points are unclassified; the yellow points are in the same cluster as the seed; and the black points are in a different cluster.

**Figure 2:**
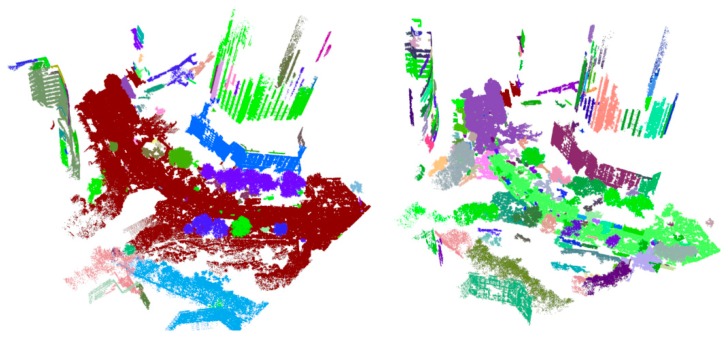
Segmentation results of MLS based on region growing. **Left**: Incomplete segmentation (Roads and trees are classified as belonging to the same class); **right**: Excessive segmentation (The same types of elements, such as roads, trees, and buildings, are classified as belonging to multiple classes).

Region growing is mainly used for point cloud segments, especially those of buildings. The 3D building model can be constructed on this basis. For a single type of building, the types of clusters are consistent, and the cluster difference is more obvious, because artificial buildings are mutated. Therefore, the method of region growing for building segments is effective. However, urban point cloud data from MLS not only have a huge number of points but are also composed of many types of geographical objects, such as roads, vegetation, and buildings. Among these, the road spatial distribution is similar to a horizontal plane, the building is similar to a vertical plane, and the vegetation is similar to 3D space agglomeration. The other types are irregular. Thus, it is difficult to use a unified standard in the region growing method. Even with a unified standard, in contrast to the mutation of buildings, there are many terrain gradients in urban areas. In addition, local features, such as a normal curvature, are often used in region growing. However, by using local features, the segment results of MLS are not satisfactory, because they are noisy. Thus, many questions have arisen about incomplete or excessive segmentation, when region growing is used for segmentation with MLS (Figure 2).

### 2.2. Urban Road Extraction Based on Region Growing

A road is a single geographical element, and the spatial locations of road points are similar, being distributed on a relatively flat surface. While it is difficult to classify all types of elements using uniform criteria in region growing, this can aid in the extraction of road pavement, due to the similarity of road points. In region growing, there are two factors that are important in the extraction of urban roads: the selection of seed points and the determination of urban road clusters. These two factors both determine whether the road surface continues to spread. In this research, we propose an automated method of road extraction, based on region growing, using MLS data, including the selection of the initial seed, decision on road point growth, new seed selection and discontinuous road strategy.

#### 2.2.1. Selection of the Initial Seed Point

The initial seed point is the most important; it determines the starting position of the road. Based on region growing, our concept did not consider all points from start to finish, but as many points on the road as possible. This reduces the computing time. Therefore, the initial seed must be located on the road.

Common methods of choosing the initial seed depend on the minimum elevation or curvature. For the elevation, the point of minimum elevation may be on the road under normal circumstances, but there are exceptions. For example, gross error points below the road or points in concave areas are most likely to be the initial seed, according to the rule of elevation. Thus, the initial seed point, determined through a search for the lowest elevation, is probably not located on the road. The curvature describes the curvature of a surface. Generally, the surface of the road does not bend significantly, but there are many flatter surfaces in the MLS dataset, e.g., building façades, billboards, and buses. In addition, the number of points in the MLS dataset is huge, and the description of the curvature is only a single value, which may cause many more than one point to have the same minimum curvature. Consequently, using the point with the smallest curvature as the initial seed cannot ensure that it is located on the road.

In this paper, we propose a method of selecting the initial seed point based on the number of neighbors, curvature, and elevation. First, the initial seed must be located on the road, and the points on the road must be distributed densely and uniformly. Thus, the number of neighbors around the initial seed should be sufficient. A portion of the points are not considered if the number of neighbors is less than a certain threshold. This is shown in Equation (1), where *P*_0_ is the seed, $N_{P_{0}}$ is the number of neighbors, and *σ_Nn_* is the threshold. In Equation (1), as in Equation (2), the neighborhood radius is set to four times the sampling distance, and *σ_Nn_* is five. The point with the smallest curvature is regarded as the initial point (Equation (2)), and if the number of points with the smallest curvature is more than one, the initial point should be that with the lowest elevation among the points with the same smallest curvature (Equation (3)). The intent of Equation (3) is to avoid points not on the non-road becoming a seed.

(1) $$N_{P_{0}}>\sigma_{Nn}$$

(2) $$K_{P_{0}}=\min\left(\left|K_{i}\right|\right),\;i=1,2,\cdot \cdot \cdot ,n$$

(3) $$H_{P_{0}}=\min\left(H_{j}\right),\;j=1,2,\cdot \cdot \cdot ,m$$

In Equations (2) and (3), $K_{P_{0}}$ and $H_{P_{0}}$ are the curvature and elevation of the seed, respectively; *K_i_* is the curvature of the *i*-th point, *n* is the number of points in a dataset, *H_j_* is the elevation of the *j*-th point, and *m* is the number of points with the same minimum curvature. The curvature has positive and negative values. Here, we need only consider its magnitude, without regard to the positive and negative values, so the absolute values of the curvature are used to determine the initial seed. The determination is conducted in a sequence. The number of neighbors is determined first, followed by the curvature and, finally, the elevation. If a point is not satisfied in the previous condition, it is directly ignored, without considering the subsequent conditions. This can improve the efficiency of the method.

There are several different types of curvature, the principal [36,37], mean [38,39], and Gaussian [39,40]. The principal curvature includes the maximum and minimum curvature. There are an infinite number of orthogonal curvatures at a point on the surface where there is a curve, such that the curvature at this point is the maximum (maximum curvature), and the curvature perpendicular to the maximum curvature surface is the minimum (minimum curvature). The mean curvature is the arithmetic mean of two principal curvatures (Equation (4)), and the Gaussian curvature is the product (Equation (5)).

(4) $$K_{m}=\left(K_{\max}+K_{\min}\right)/2$$

(5) $$K_{g}=K_{\max}\cdot K_{\min}$$

In the above formulae, *K*_max_ is the maximum curvature, *K*_min_ is the minimum curvature, *K_m_* is the mean curvature, and *K_g_* is the Gaussian curvature.

The road pavement is an approximate plane for considering the road point from the curvature value, following Equation (1). However, in addition to the road, there are other elements in the urban point cloud dataset that may be closer to the plane, for example, a billboard. In order to avoid an erroneous selection of points on a billboard, instead of the road points in Equation (2), it is necessary to reduce the difference in curvature between the points on the road pavement and on the other plane as much as possible.

Road pavement is not an absolute plane and is more undulating than a billboard. In terms of curvature, the maximum and minimum curvature of the points on the billboard are theoretically close to zero. For the road points, the minimum curvatures are also approximately equal to 0. However, the maximum curvatures are higher than the point on the billboard. If the maximum curvature is used in Equation (2), the result of the selection is definitely a point on the billboard, and the average curvature is the same. Moreover, if the minimum curvature is chosen, the number of points that satisfy Equation (2) will greatly increase. This will increase the computational expense of Equation (3). More importantly, the results of the initial seed point are often incorrect. Once there is a point where the minimum curvature is close to 0, and the elevation is below the point on the road, the results will be incorrect. The Gaussian curvature neutralizes the maximum and minimum curvatures. The road surface is relatively flat, and the difference between the maximum and the minimum curvature is not large, so the Gaussian curvature of the points on the road is close to 0. Other points with a minimum curvature of 0 have a Gaussian curvature higher than the road points, because they are not on a flat surface, and their maximum curvatures are significantly higher than the minimum.

**Figure 3:**
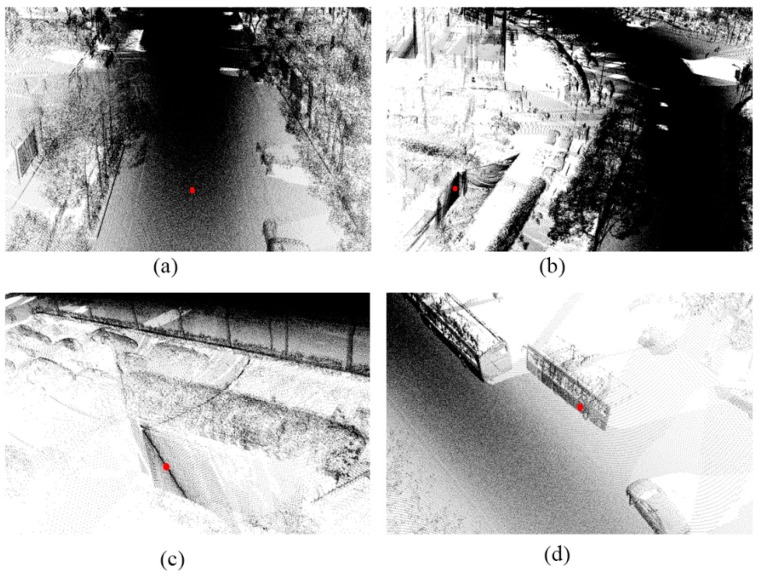
Initial seed point with different curvatures: (**a**) Gaussian curvature; (**b**) mean curvature; (**c**) maximum curvature; (**d**) minimum curvature.

Figure 3 illustrates the results of the initial seed for an MLS dataset using various curvatures. From the figure, the initial seed with Gaussian curvature is located on the road, whereas the other three curvatures are on the vertical surface of an object, such as a billboard or bus. Thus, the Gaussian curvature has a better stability, and the probability that the initial seed will be on the road is greatly increased using Equation (3) with Gaussian curvature.

#### 2.2.2. Decision on the Growing Condition

Once each seed of the road is determined, its K neighbors are considered in terms of whether they belong to the same class as the road. The principle for determination is a similarity of spatial or geometric attributes. If that similarity is strong, the neighbor will be classified as a road. While the curvature describes the spatial properties of the point, it is too rough to distinguish between the classes of road and non-road from a huge number of points. Therefore, it is necessary to use a new criterion, with a greater distinguishing capacity, to extract the road.

**Figure 4:**
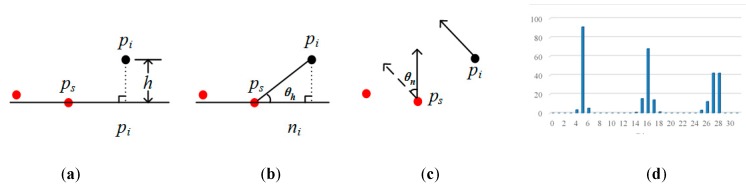
The principle of different methods in the judgment of the cluster belonging to an urban road, (**a**) difference in elevation (DE): *h* is the height of *p_i_* at the level of the seed; (**b**) horizontal angle (HA): *θ_h_* is the angle of *p_i_* at the level of the seed; (**c**) angle between two normal points (AN): *θ_n_* is the angle of *p_i_*, relative to the tangent plane of the seed; (**d**) Fast Point Feature Histograms (FPFH): The abscissa represents the subinterval of the angle in three directions, and the ordinate is the percentage of the number of points.

Currently, common methods are based on a difference in elevation [41] (hereafter referred to as DE), horizontal angle [42] (referred to as HA), the angle between two normal points [43] (referred to as AN), the point feature descriptor, etc. The principles are shown in Figure 4, where *p_s_* is the seed point, and *p_i_* is a neighboring point. The methods of DE and HA treat the urban road as a horizontal plane, but, in fact, the law is such that the road center is slightly higher than the edge, and some roads have a certain slope. AN calculates the angle between the normal points of the seed and its neighbor. The normal point is affected by the search radius, and when vegetation is connected to the road, its normal points may be consistent with the road. Descriptors aim at encoding the local shape around a point in terms of a set of numerical values, the vast majority of which are in the form of high-dimensional histograms. By calculating the Euclidean distance between histograms of the seed and its neighbors, the difference between points can be obtained. For descriptors, Fast Point Feature Histograms [44] (referred to as FPFH) have a good overall performance in terms of efficiency, robustness and descriptiveness (Figure 4d). However, descriptors consider differences in the spatial or geometric attributes of all points and do not aim at a particular type of geographic element, such as a road. Therefore, descriptors do not have an advantage in extracting roads.

**Figure 5:**
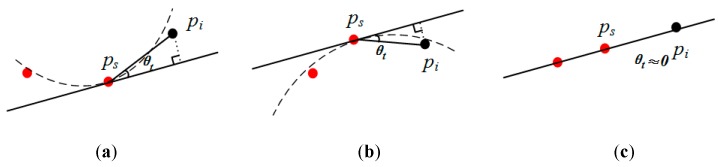
The principle Tangent plane angle: (**a**) the concave; (**b**) the convex; (**c**) the terrain with a stable slope.

Our proposed method for determining the road class in order to extract roads from an MLS dataset is shown in Figure 5. While one needs to calculate the angle, in contrast with HA, the angle between the tangent plane of the seed and the connection between the neighbor and seed (referred to as TA) are used for determination.

Equation (6) is used to calculate TA, where *θ_t_* is the value of TA, *n* = (A, B, C) is the normal vector of the tangent plane, (*x_s_*, *y_s_*, *z_s_*) is the 3D coordinate of the seed, and (*x_i_*, *y_i_*, *z_i_*) is the *i*-th neighbor of the seed. The criterion for determining whether a point belongs to the road class is represented in Equation (7), where *p_i_* is the *i*-th neighbor of the seed, and *σ_A_* is the threshold of TA. If *θ_t_* is ≤ *σ_A_*, the point belongs to the road (and vice versa).

(6) $$\theta_{t}=\arcsin\left(\frac{\left|A(x_{s}-x_{i})+B\left(y_{s}-y_{i}\right)+C\left(z_{s}-z_{i}\right)\right|}{\sqrt{{\left(x_{s}-x_{i}\right)}^{2}+{\left(y_{s}-y_{i}\right)}^{2}+{\left(z_{s}-z_{i}\right)}^{2}}}\right)$$

(7) $$p_{i}=\{\begin{array}{c} true,\;\theta_{t}\leq \sigma_{A}\;\\ false,\;\theta_{t}>\sigma_{A}\; \end{array}$$

When the road is similar to a horizontal plane, HA and TA are nearly the same. If not, the difference is greater. Figure 5 illustrates three different situations: panel (a) for the concave, (b) for the convex, and (c) for the terrain with a stable slope. It is seen that, for a sunken road, the value of TA is less than that of HA, whereas, for a raised road, the contrary is the case. While there are greater and lesser TA values, larger angles are reduced for a sunken road. When the terrain has a stable slope, the values of TA, which are similar to those of HA on a horizontal plane, become very small. Therefore, based on TA, the range of angles on the road are smaller, which makes it easier to distinguish between the road and other elements.

#### 2.2.3. Determination of New Seeds

The growth of roads is carried out by constantly adding new seeds. These seeds are used to control the spread from the initial seed to the entire road. Once a new seed is confirmed, it can be used to determine whether its neighbors belong to the road by searching for neighboring points. Thus, new seeds have a great impact on the accuracy of road extraction. Equation (8) shows the principle of the new seed:

(8) $$p_{i}=\{\begin{array}{l} true,\;p_{i}\in C_{r}\cap K_{p_{i}}<\sigma_{K}\;\\ false,\;otherwise\; \end{array}$$

where $K_{p_{i}}$ is the Gaussian curvature of a point *p_i_*, *σ_K_* is the threshold of the Gaussian curvature, and *C_r_* is the collection of road points. The determination of new seeds depends on two criteria: the same cluster as the seed and the Gaussian curvature. If a neighboring point of the previous seed is identified as the road cluster, it may be a seed, because it has passed the validation, conducted using Equation (7). Otherwise, this point is certainly not a seed. However, this is not enough. When a point is located on the edge of the road, it is no longer suitable to be a seed. This situation can be filtered using Gaussian curvature. Since the edge of the road is an artificial mutation, the Gaussian curvatures of these points are significantly larger than the road points. Therefore, the points at the edge can be detected by setting a threshold for the Gaussian curvature, in order to determine whether region growing continues. Therefore, the second criterion is that the Gaussian curvature of the point must be less than a certain threshold.

#### 2.2.4. Strategy of Discontinuous Roads

Following the method presented above, the road can theoretically be extracted accurately, if the seed selection and road determination are correct. There is one exception: when there is more than one road in a point cloud dataset, which is not contiguous with another, only one road can be extracted. This method of region growing is implemented via the constant spread of points. Thus, when the first road is extracted, there is no longer a new seed and point belonging to the road that meets the conditions, because of the discontinuous area between the roads.

According to the method presented, the initial seed must be located on the road. When there is more than one road within a point cloud dataset, our method is still applicable to the remaining points, even if one road is extracted. That is, for the remaining points, the initial seed, re-determined by Equations (1) to (3), must be on another road. Thus, to extract all discrete roads, the remaining points can be recalculated using the method of selecting the initial seed and determining the points belonging to the road (Section 2.2.1 and Section 2.2.2).

**Figure 6:**
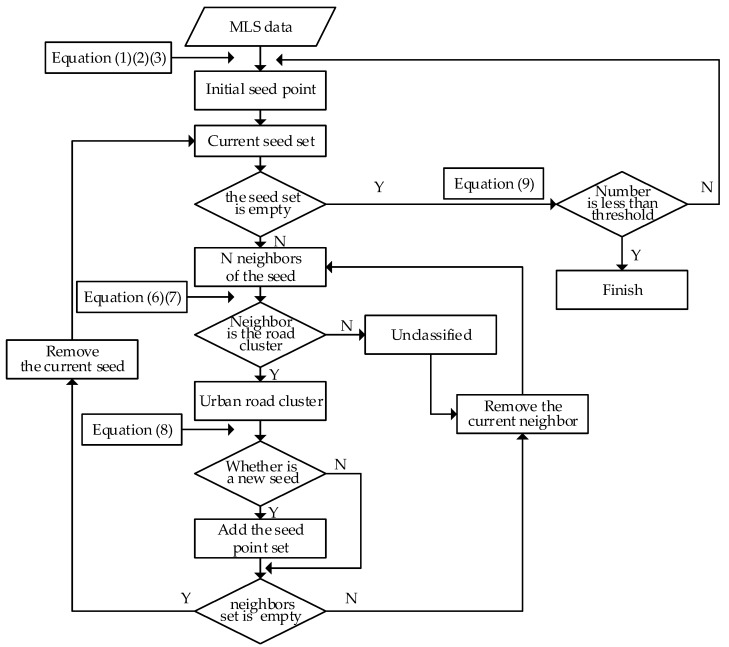
Flowchart of automatic urban road extraction with region growing.

The key to the method is when region growing ends. The normal method based on region growing constantly repeats the search for new initial seeds, and extracts points belonging to the initial seed, until all points are classified. Afterward, all classes are identified in terms of whether they belong to the road. However, the efficiency of this method is low, because it needs to traverse all points. As mentioned earlier, all road points will be extracted first, and then the other points can be extracted. The number of road and non-road points varies widely, and the number of points on the road is much greater than that of other types. Therefore, we can set a threshold for distinguishing between roads and non-roads. Equation (9) shows the basis for this determination:

(9) $$\left\{ \begin{array}{l} Roads=\left\{C_{i}|i=1,2,\cdot \cdot \cdot ,k-1\right\}\\ N_{C_{k}}<\sigma_{N_{r}} \end{array}\right.$$

where *C_i_* is the *i*-th class, $N_{C_{k}}$ is the number of points of the *k*-th class, and $\sigma_{N_{r}}$ is the threshold. When the number of points of the *i*-th class exceeds the threshold, the current class is considered a road, and region growing continues. Otherwise, the current class is a non-road, and the algorithm stops. The final roads are all the classes, before the current one. Figure 6 illustrates the algorithm flowchart of urban road extraction, with region growing.

#### 2.2.5. Search Strategy for Neighboring Points

Searching for the points nearest to a given point is often involved in 3D point cloud data processing, such as in the present calculation of normal points and curvature and region growing. The search strategy depends on the data structure of the point cloud, and there are two main types: the K-D tree and octree. The K-D tree is used for searching for neighbors and has two means for determining the neighbors. One is to search for the number of the neighborhood (N neighborhood), and the other is to search for the radius. While they are all based on the K-D tree structure, there are some differences between the two. N neighborhood searches for the nearest k points from the current point, whereas the method based on the radius searches for all points within a sphere, of which the current point is the center, and *r* is the radius.

When calculating the normal points and curvature, the values are influenced by the neighbors. If the number of neighbors is too small, the values may be NaN. Therefore, N neighborhood is typically used for normal points and curvature, because it ensures that the number is always k. However, when extracting the road using region growing, the N neighborhood and radius search methods are both flawed. If, when the radius is used for searching for the nearest points, it is too small, the region may be unable to grow to the entire road. If it is too large, many non-road points may be misjudged near the boundary. Furthermore, it is difficult to find a suitable radius value to achieve a high accuracy of road extraction, because the resolution, nearest distance, and road width in different data are not the same. When N neighborhood is used, the neighbors may contain outliers, discrete or sparse points at the boundary, which may be considered as road points. In addition, the problem of finding the threshold for the optimum accuracy is the same as in the search radius.

To improve the applicability and robustness of our method, N neighborhood and search radius were both used to constrain the proximity points. That is, the radius was set to a larger value for searching for neighbors, and an upper limit was used to control the maximum number of points. Thus, there is no case in which the road points cannot be completely traversed because the radius is too small, and a large number of non-road points are determined, even if the radius is too large. Detailed results are presented in Section 4.3.2.

## 3. Experimental Setup

### 3.1. Test Data

Five data are used to test the proposed method, namely, a simple road, partially sheltered road (referred to as a sheltered road), partial continuous road with an isolation belt (referred to as a partial continuous road), discontinuous road with an isolation belt (referred to as a discontinuous road), and multiple roads of different types (referred to as multiple roads). All data were acquired by MLS in 2015, and the data area is the city of Nanjing. The number of points in each dataset is between 3 and 8 million, and the nearest distance of the points is 0.02 m.

**Figure 7:**
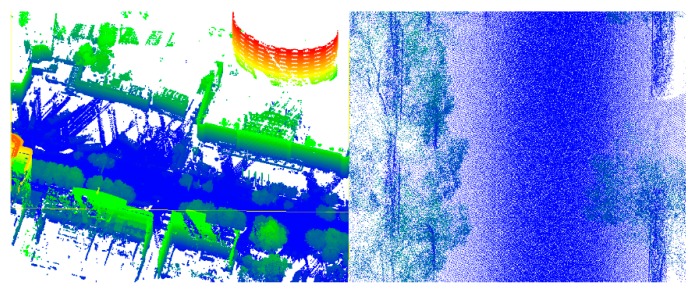
Simple road data. **Left**: global map; **right**: partial enlarged view.

The most homogeneous data were on the simple road. There is no shelter or isolation belt on the road, and the road is continuous and straight. However, the curb is discontinuous. The simple road data are shown in Figure 7.

**Figure 8:**
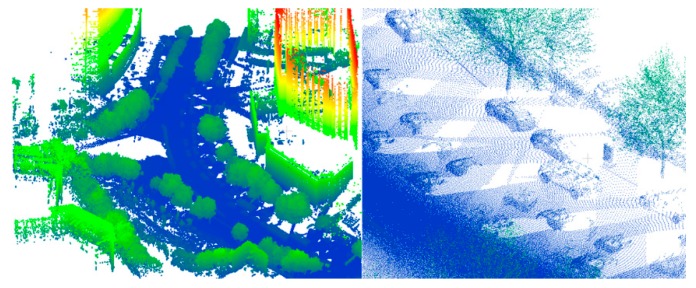
Sheltered road data. **Left**: global map; **right**: partial enlarged view.

The sheltered road has numerous occlusions, because there were many vehicles on the road at the time of data collection. For this type of road, there are many blank areas in the data, but the points on the road are connected overall. The purpose of using the sheltered road is to test whether a road with occlusions can be effectively identified. In addition, the sheltered road data are curved, which can be seen in Figure 8.

**Figure 9:**
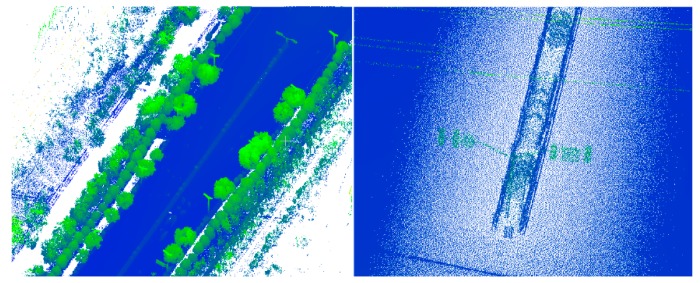
Partial continuous road data. **Left**: global map; **right**: partial enlarged view.

The partial continuous road (Figure 9) is also straight, but, in the middle of the road, there is an isolation belt that does not completely separate the road. At the end of that belt, the two-way road is continuous. The purpose is to test whether our method can effectively avoid the isolation zone and spread from the connected area.

**Figure 10:**
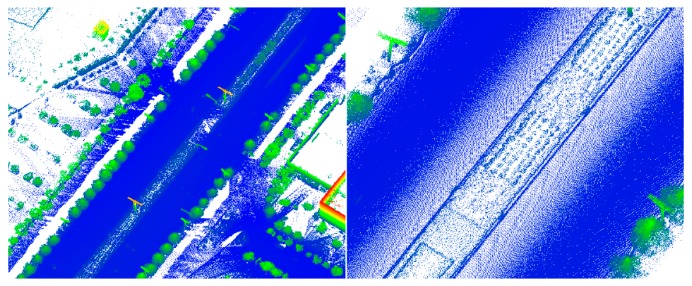
Discontinuous road data. **Left**: global map; **right**: road profile view.

The discontinuous road data are similar to those on the partial continuous road. The difference is that the two-way road is discontinuous (Figure 10). From the profile view, the elevations of the points on the isolation belt are all greater than those on the road. The purpose is to test the effect of the algorithm when applied to discontinuous roads.

**Figure 11:**
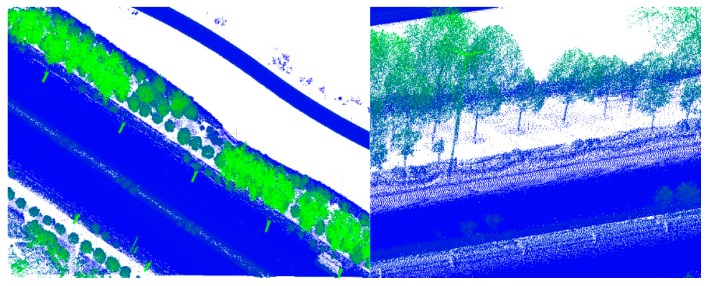
Multiple roads data. **Left**: global map; **right**: road profile view.

The data on multiple roads are different from the previous data. In these data, not only is one road completely separated from the isolation belt, but there are also other types of roads. As shown in Figure 11, there is a non-motorized road beside two discontinuous roads. Additionally, a large number of points are located below the road. The purpose is to test whether the three initial seeds are accurately located on three types of roads, and whether we can achieve a high accuracy for different types of roads.

There are other differences. All data have outliers, and those on multiple roads are the most numerous, followed by those on the simple road, sheltered road, partial continuous road, and discontinuous road. Many of the outliers in the different data are below the roads and can be used to verify the effect of selecting the initial seed, using our proposed method. Details of these data are listed in Table 1.

### 3.2. Ground-Truth Road

**Figure 12:**
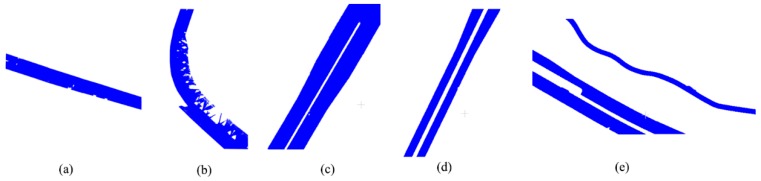
Ground truth: (**a**) simple road; (**b**) sheltered road; (**c**) partial continuous road; (**d**) discontinuous road; (**e**) multiple roads.

Ground-truth roads are used for calculating accuracy. Because of the boundary and continuity of the road, it is easy to distinguish between a road and non-road by artificial judgment. Ground-truth roads for the five data were manually selected along the curb of the road using CloudCompare software. However, in practice, not all curbs are continuous, and many are broken. This causes some trouble when selecting ground-truth roads. The criterion for selecting ground-truth roads is that the broken curbs are connected with a closed section along their edge lines, while maintaining the overall shape of the curb line. The points within the closed area default to ground-truth roads, and the other points are deleted. The results for the ground-truth roads are shown in Figure 12.

### 3.3. Accuracy Evaluation

We extracted roads from five data using our TA method. The other four methods (DE, HA, AN, and FPFH) were also implemented for the same data to compare the results. The comparative analysis includes accuracy, sensitivity, and the influence of other parameters.

#### 3.3.1. Accuracy Evaluation Criteria

We used the Kappa coefficient to evaluate the accuracy of the results for the extracted roads and ground-truth roads. Kappa is an important index for evaluating the consistency and reliability of classification results [45]. It is commonly used in the evaluation of classification. Kappa is also suitable for assessing the results of geographic element extraction. The formulae of Kappa are as follows:

(10) {k=PA−Pe1−PePA=(a+d)nPe=(a1⋅b1+a0⋅b0)n2

where *k* is the value of Kappa, *P_A_* is the observed agreement, *P_e_* is the chance agreement, *a*_1_ is the ground-truth roads, *b*_1_ is the extracted roads, *a*_0_ is the ground-truth non-roads, and *b*_0_ is the extracted non-roads. The larger the Kappa value, the greater the precision.

#### 3.3.2. Sensitivity to the Road Threshold

While Kappa reflects accuracy, a variable threshold *θ_t_* produces a variety of accuracy values. This is because, in urban road extraction based on region growing, some points are considered to belong to a road with a small threshold but not a larger threshold.

To demonstrate these rules, we experimented with the five methods (TA, DE, HA, AN and FPFH) and five data. In each method, 20 threshold values, ranging from 0.005 to 60, were used. The total number of experiments was 500, and the results were shown by broken line graphs. While the units of the thresholds in each method were inconsistent (degree, distance and others), the main purpose of the experiment was to investigate the overall sensitivity of those methods.

#### 3.3.3. Influence of Gaussian Curvature and the Search Radius

There are other parameters that affect the results of urban road extraction. The two most important are Gaussian curvature and the search radius. Gaussian curvature is used to determine whether a point is a seed. The larger the threshold of the Gaussian curvature, the higher the probability of becoming a seed, and the greater the number of points considered to be a road. Nevertheless, the effect of different thresholds on accuracy is unclear, which is also the case for the search radius.

Thus, we performed two sets of experiments, one for the threshold of the Gaussian curvature *K_g_* and the other for the search radius *r*. In each set of experiments, we used six thresholds, ranging from small to large.

## 4. Experimental Results and Analysis

### 4.1. Results of Road Extraction

**Figure 13:**
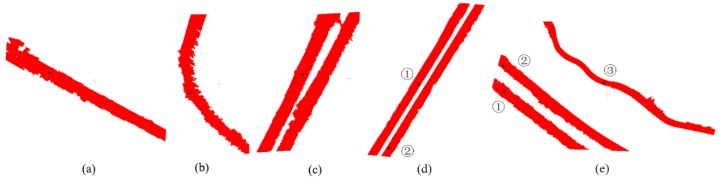
The results of road extraction, the numbers ①, ② and ③ in the figure indicate the numbers of independent roads: (**a**) simple road; (**b**) sheltered road; (**c**) partial continuous road; (**d**) discontinuous road; (**e**) multiple roads.

The results of urban road extraction from the five data are shown in Figure 13, in which panels (a) to (e) are the simple road, sheltered road, partial continuous road, discontinuous road, and multiple roads, respectively. The black points in the figure are the initial seeds, and the red points are the urban roads. The numbers in (d) and (e) are the serial numbers of the seeds from different discontinuous roads.

**Figure 14:**
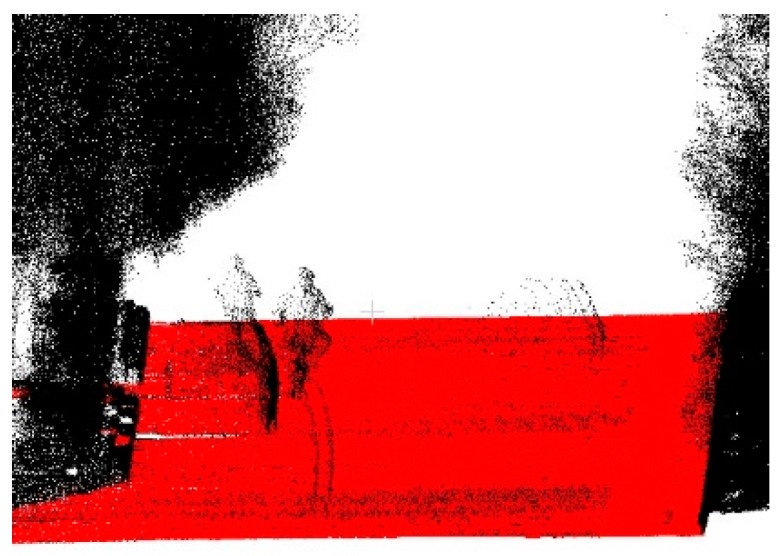
Visualization of extracted road overlay MLS data: the red points belong to the extracted road and the black are the non-road points.

As shown in Figure 13, all the initial seeds from the different data were on the roads. When the number of roads is greater than 1 in a datum, each initial seed is located on a different road. This indicates that the method of selecting the initial seed for different roads was correct. Figure 14 shows the visualization of the extracted road overlaying MLS data. It can be seen that points above the road surface (vehicles, pedestrians, etc.) can be clearly distinguished. At the same time, the extracted road has very high precision at the curb, but when the curb line is discontinuous, some points at the break will be misjudged.

Table 2 shows the accuracy of the five data. Overall, the roads extracted from the five data are accurate, as all have an accuracy of over 90%. This shows that the method proposed in this paper is feasible and can achieve good results. Specifically, the accuracies of the discontinuous road and multiple roads are the highest, exceeding 94%, followed by the continuous road, which exceeded 92%. The accuracies of the simple road and occluded road were the lowest, exceeding 90%.

In the five data, the curb and intermediate barrier of the discontinuous road and multiple roads are continuous, and both have obvious boundaries. The partial continuous road also has a continuous curb and intermediate barrier, but the boundary of the isolation zone is not as obvious as it was in the first two roads. The curbs of the simple and sheltered road are discontinuous and have many breaks. It can be seen, from Figure 13, that the simple and sheltered roads with the lowest accuracy have some serrations on the curb, while this situation does not appear in the discontinuous road and multiple roads. The partial continuous road falls somewhere in between. Therefore, the method in this paper can achieve good results on the whole. Nevertheless, it is sensitive to curbs and depends on the continuity and clarity of curbs.

### 4.2. Comparison with the Accuracy of Other Methods

**Figure 15:**
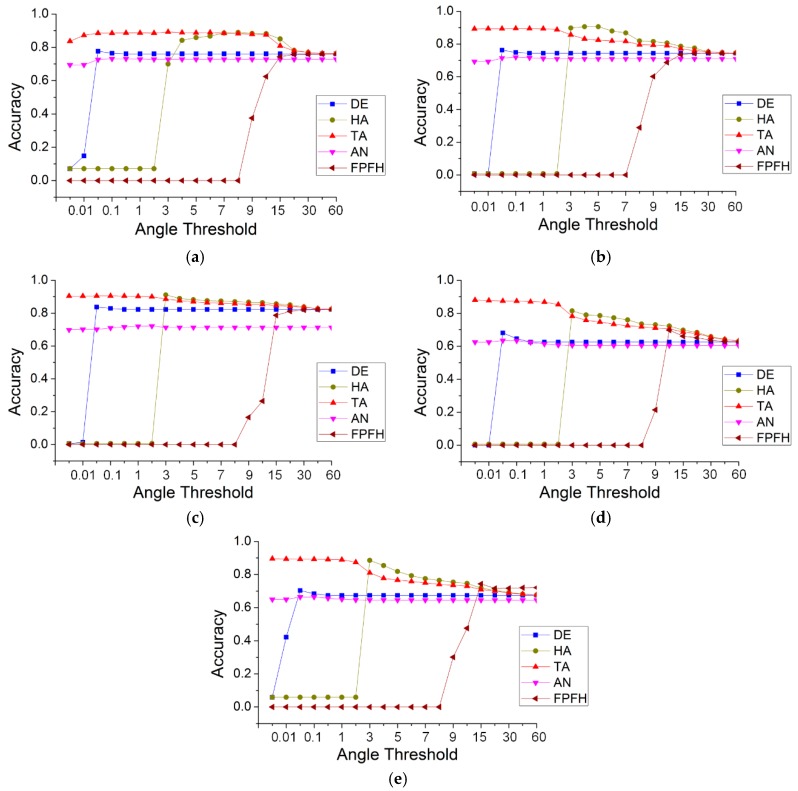
The accuracy of the five methods, with different thresholds, for the five data: (**a**) simple road; (**b**) sheltered road; (**c**) partial continuous road; (**d**) discontinuous road; (**e**) multiple roads.

To compare the accuracy of TA with that of the other methods, we conducted five sets of experiments, in which the thresholds were uniformly set to 20 different values: 0.005, 0.01, 0.05, 0.1, 0.5, 1, 2, 3, 4, 5, 6, 7, 8, 9, 10, 15, 20, 30, 45 and 60. While the threshold units of the methods were inconsistent, the purpose of the experiment was to determine the range and stability of the accuracy of those methods. The values of the other parameters remained the same, i.e., the threshold of the Gaussian curvature was 0.02, the search radius was 0.24, and the maximum number of neighborhoods was 30. Figure 15 show the results of the different methods for the five data.

As shown in Figure 15, for simple roads, the greatest accuracies of TA and HA were optimal and nearly identical, followed by those of DE and FPFH, and those of AN were the lowest. The minimum accuracy of TA was the highest, followed by that of AN. The other three methods had the lowest minimum accuracy. This behavior was the same for the other four datasets. However, with an increase in the threshold, the TA method always achieved a high accuracy (close to 90%), until the angle threshold was 15. The accuracy of DE was very low (less than 10%), prior to the threshold reaching 0.01; beyond that value, the accuracy was stable but below 80%. This indicates that, when the height difference between neighboring points was less than 0.01 m, DE could not correctly distinguish between road and non-road. Even if the accuracy of DE was stable, it was low. HA could not recognize road points when the horizontal angle threshold was less than three degrees, and, like TA, its accuracy began to decline when that threshold was greater than 15 degrees. This reveals that the effective distinction between horizontal angles was greater than three degrees. AN could identify road points at a very small threshold of the normal angle difference, and its accuracy was very stable but too poor. This indicates that the normal angle difference between neighbors was very small and could not be used to effectively distinguish between road and non-road points. FPFH represents the feature descriptor of a point, which distinguishes its characteristics by calculating the Euclidean distance of the histogram. FPFH can recognize road points only if the difference threshold of the Euclidean distance is greater than eight. In addition, the highest accuracy it can achieve is the same as the minimum accuracy of TA. Thus, the point feature descriptor is not the best method for identifying road points.

Because of the obvious shortcomings of the DE, AN and FPFH methods, only the TA and HA methods were subsequently analyzed in relation to the other four data. For the sheltered road, HA attained a high accuracy (between 3 and 7), while that of TA was less than three. For a threshold ≤3, TA showed obvious advantages. For a threshold >3 and <8, the accuracy of HA was greater than that of TA, but the difference was less than 10%. For the remaining three data, the behavior of HA and TA was more similar. For a threshold less than three, HA could not recognize the road, whereas TA achieved a high accuracy. For a threshold ≥3, the accuracies and trends of HA and TA were roughly the same. Table 3 shows the accuracy range of the five methods and five data.

From Table 3, the minimum accuracies of TA for the five data were all optimal, with the greatest accuracies for the simple road, discontinuous road and multiple road data. For the other two data (sheltered road and partial continuous road), HA achieved the greatest accuracies, but the accuracy difference between TA and HA was less than 10%. Table 4 shows the accuracy distribution of the five methods.

From Table 4, it can be seen that, among all the experimental results of this section, the number of accuracies >90% for the TA method was six; HA produced three; and the other produced zero. The number of accuracies between 80% and 90% using TA was 57, which is much greater than that of HA (30) and the others. The number of accuracies >80% using TA was 63, accounting for 63% of the total, whereas the highest proportion was only 33% using the other methods. These results include some large and obviously unsuitable thresholds. If some larger thresholds were removed, the proportion of TA increased. Thus, the proposed method of TA could attain a high accuracy. More importantly, such a high accuracy could always be maintained within a threshold interval that is common in practical applications. For all five data, the angle range at which a high accuracy was maintained, was 0.01 to 3 degrees.

### 4.3. Influence of Parameters

Different values of parameters will cause the accuracy of the results to be different. This section discusses the influence of parameters on accuracy and finds the threshold ranges of the parameters that can be obtained with a high precision.

#### 4.3.1. Gaussian Curvature

**Figure 16:**
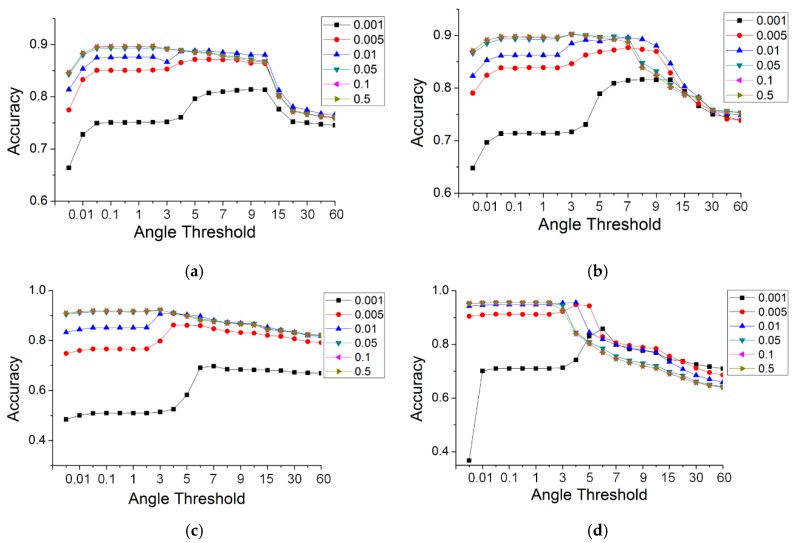

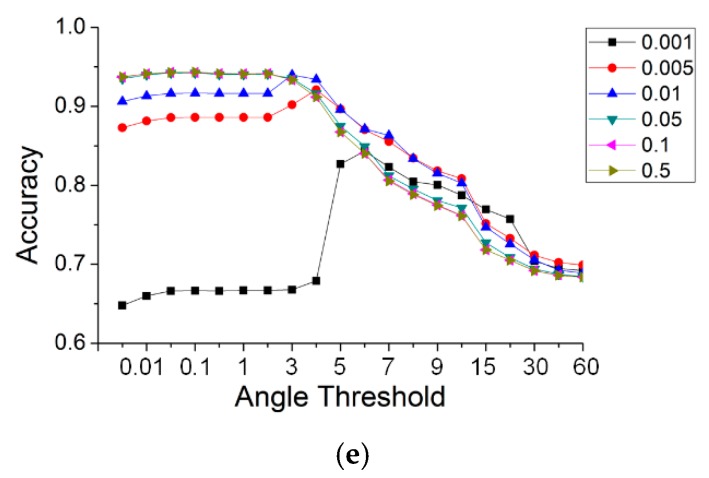
The accuracy of TA, with different thresholds of curvatures and angles, for the five data: (**a**) simple road; (**b**) sheltered road; (**c**) partial continuous road; (**d**) discontinuous road; (**e**) multiple roads.

Gaussian curvature was used for the selection of seeds in road extraction. Whether or not the seeds are correctly selected had an effect on the accuracy of road extraction. To ascertain the relationship between the curvature threshold and accuracy, another set of experiments with the TA method were run for the five datasets, in which the curvature thresholds were set to 0.001, 0.005, 0.01, 0.05, 0.1 and 0.5. The angle thresholds were the same as those used in Section 4.2. Figure 16 illustrates the results for the different curvature thresholds. Some curves with curvature thresholds are not visible in Figure 16, e.g., curves with thresholds of 0.05 and 0.1 in the simple road dataset. This is because those curves are obscured by curves with a larger curvature threshold. This indicates that the results are robust when the value of the Gaussian curvature rises by a specific value.

As shown in Figure 16, the change laws of all of the data are similar. The accuracies increased greatly with the value of the Gaussian curvature, and this rule was especially noticeable when the value of the TA was small. When the curvature value rose to a specific value, the curves tended to become stable and coincide at a particular location. This indicates that increasing the curvature threshold can improve the accuracy. Further, the change in the curves produced a convergence. For a curvature threshold >0.05, the accuracies remained constant and were no longer affected by that threshold. Therefore, a larger curvature threshold, which can not only achieve a high accuracy but maintain it over a large angle range, has versatility in practical applications.

On the whole, the threshold of the Gaussian curvature had an effect on the accuracy of the results, which increased as the curvature threshold increased. At the same time, when the curvature threshold was increased to a specific value, the results converged. For the most accurate results, there were general threshold ranges, i.e., the value of Gaussian curvature was greater than 0.05, and the angle of the TA was between 0.01 and 3 degrees.

#### 4.3.2. Neighborhood Search

**Figure 17:**
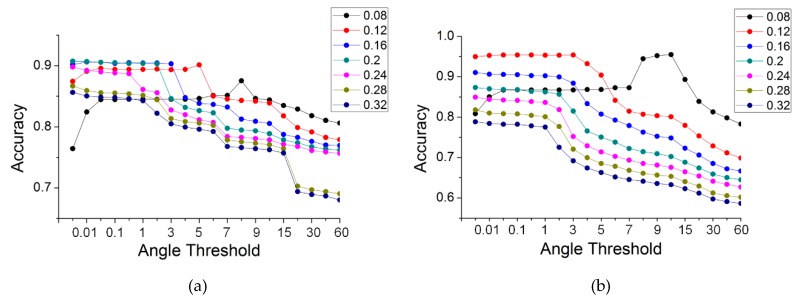
The accuracy of TA, with different search radii, for two of the datasets. (**a**): simple road; (**b**): discontinuous road.

The search radius had an effect on the accuracy of the results. To clarify the specific effects, the search radii were set to 2, 4, 6, 8, 10, 12, 14 and 16 times the distance of the neighbors (the distance of the neighbors was 0.02 m). The partial results of the five data were similar, so Figure 17 shows only the results for two of the datasets: panel (a) is for the simple road; and (b) is for the discontinuous road.

**Figure 18:**
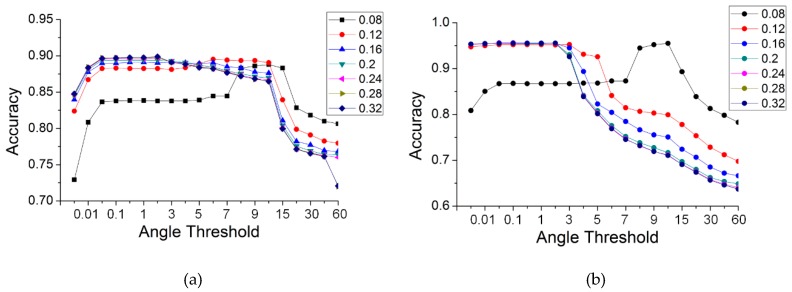
The accuracy of TA, with different search radii and thresholds, for the five data. (**a**): simple road; (**b**): discontinuous road.

As shown in Figure 17, the variation in the precision with different radii in the two data was somewhat similar. For a small angle threshold (less than three degrees), the accuracies first increased and then decreased with the increase in the search radius. For a large threshold (more than six degrees), the accuracies always decreased. However, the behavior of the results for the two data was not identical. To improve the versatility and robustness of the proposed method, the technique advanced in Section 2.2.5 was used for neighboring point searching, i.e., the radius and maximum number of neighbors were used together. We conducted several other sets of experiments. Among these, the search radii were set as before, and the maximum number of neighbors was set to 30. Similarly, Figure 18 illustrates the results for two of the datasets: panel (a) is for the simple road; and (b) is for the discontinuous road.

As shown in Figure 18, the results for the two datasets are very similar. With an increasing radius, the accuracies for a smaller angle threshold (less than two degrees) increased and gradually converged. When the radius reached a specific value, the accuracies maximized and stabilized. However, there is a slight difference. The radius with stable accuracies was 0.24 m for the data on the simple road, sheltered road and partial continuous road; 0.16 m for the data on the discontinuous road; and 0.20 m for the data on multiple roads. By selecting the intersection of the parameter thresholds for different results, it can be found that there are also general threshold ranges for the best results, i.e., the search radius is more than 12 times the sampling distance (0.24 m in this paper), and the maximum number of neighbors is 30.

#### 4.3.3. General Parameter Range

Table 5 shows the optimal accuracy range for the uniform parameters in the five data.

As shown in Table 5, for a curvature greater than 0.05, a search radius greater than 0.24 m, a maximum number of neighbors of 30, and an angle threshold greater than 0.01 and less than three degrees, the maximum accuracies of the five data were all greater than 90%. For the lowest accuracies, the differences from the maximum were less than 0.016. This shows that the present method not only can achieve a high accuracy in extracting roads but also has a large range of parameters. Consequently, the TA method is stable and robust.

## 5. Conclusions

In this paper, we propose an automatic method for extracting urban roads using MLS data. The initial seed is selected based on constraints relating to the height, curvature and number of neighbors. The decision on the road cluster depends on the angle of the tangent plane, and the decision on the new seeds depends on the curvature and identified road points. The selection of a new seed is constrained by road cluster points and Gaussian curvature. Additionally, the method involves the strategy of multiple discontinuous roads. The principle and complete process of the method is described in detail, and it is validated using five different types of urban road data, which are the main types of urban road data. Moreover, the effective ranges of the parameters are also given in this paper. The method proposed in this paper is versatile. When it is used with other MLS datasets, it is only necessary to set the parameters according to the given range in order to obtain high-precision urban roads. There are two main research projects for the future. The first involves extracting curbs to fit the edge of an urban road and identifying road points under an edge constraint. The second involves extracting roads and other elements using the theory of deep learning.

## Figures and Tables

**Table 1:** Data used in road extraction.

Type of Data	The Number of Points	The Type of Road	Continuous Curb	Outlier	Occlusion	Isolation Belt
Simple Road	3,306,211	Single	No	Small	Small	No
Sheltered Road	3,027,780	Single	No	Small	Large	No
Partial Continuous Road	4,658,529	Single	Yes	Small	Small	Yes
Discontinuous Road	3,663,311	Double	Yes	Small	Small	Yes
Multiple Roads	8,262,651	Multiple	Yes	Large	No	Yes

**Table 2:** The accuracy of the five data.

Data	Accuracy (%)
Simple Road	90.56
Sheltered Road	90.95
Partial Continuous Road	92.27
Discontinuous Road	96.11
Multiple Roads	94.39

**Table 3:** The accuracy range of the five methods.

		Simple Road (%)	Sheltered Road (%)	Partial Continuous Road (%)	Discontinuous Road (%)	Multiple Roads (%)
DE	Max	76.71	77.37	82.23	68.12	67.41
	Min	6.48	0.68	0.62	0.71	0.58
HA	Max	88.94	90.61	91.10	81.48	88.58
	Min	6.48	0.68	0.63	0.69	5.89
TA	Max	90.31	89.61	90.50	88.07	89.59
	Min	76.12	74.88	81.76	63.22	67.71
AN	Max	73.43	73.63	74.16	63.45	66.47
	Min	64.14	69.47	71.28	60.45	64.55
FPFH	Max	76.71	77.36	82.16	69.79	74.38
	Min	0	0	0	0	0

**Table 4:** Accuracy distribution of the five methods.

	[90,100]	[80,90]	[70,80]	[60,70]	[0,60]
DE	0	18	37	35	10
HA	3	30	24	8	35
TA	6	57	29	8	0
AN	0	0	52	48	0
FPFH	0	4	16	9	71

**Table 5:** Maximal accuracy of TA.

	The Maximum Accuracy (%)	The Minimum Accuracy (%)	Uniform Parameters
Simple Road	90.56	89.40	*K* > 0.05 *r* > 0.24 *N_max_* = 30 0.01 < *θ_t_* < 3
Sheltered Road	90.95	89.35	
Partial Continuous Road	92.27	91.19	
Discontinuous Road	96.11	95.26	
Multiple Roads	94.39	93.96	
